# Low-Dose Sevoflurane May Reduce Blood Loss and Need for Blood Products After Cardiac Surgery: A Prospective, Randomized Pilot Study

**DOI:** 10.1097/MD.0000000000003424

**Published:** 2016-04-29

**Authors:** Zhaoxia Tan, Li Zhou, Zhen Qin, Ming Luo, Hao Chen, Jiyue Xiong, Jian Li, Ting Liu, Lei Du, Jing Zhou

**Affiliations:** From the Department of Anesthesiology and Translational Neuroscience Center, West China Hospital (ZT, LZ, ZQ, ML, JX, JL, TL, LD); and Department of Laboratory Medicine, West China Hospital, Sichuan University, Chengdu, China (HC, JZ).

## Abstract

Patients undergoing cardiac surgery often experience abnormal bleeding, due primarily to cardiopulmonary bypass (CPB)-induced activation of platelets. Sevoflurane may inhibit platelet activation, raising the possibility that administering it during CPB may reduce blood loss.

Patients between 18 and 65 years old who were scheduled for cardiac surgery under CPB at our hospital were prospectively enrolled and randomized to receive intravenous anesthetics alone (control group, n = 77) or together with sevoflurane (0.5–1.0 vol/%) from an oxygenator (sevoflurane group, n = 76). The primary outcome was postoperative blood loss, the secondary outcome was postoperative need for blood products.

Volume of blood loss was 48% lower in the sevoflurane group than the control group at 4 hours after surgery, and 33% lower at 12 hours after surgery. Significantly fewer patients in the sevoflurane group lost >700 mL blood within 24 hours (9 of 76 vs 28 of 77, *P* < 0.001). As a result, the sevoflurane group received significantly smaller volumes of packed red blood cells (1.25 ± 2.36 vs 2.23 ± 3.75 units, *P* = 0.011) and fresh frozen plasma (97 ± 237 vs 236 ± 344 mL, P = 0.004). Thus the sevoflurane group was at significantly lower risk of requiring complex blood products after surgery (adjusted odds ratio [OR] 0.34, 95% confidence interval [CI] 0.17–0.68, *P* = 0.002).

Sevoflurane inhalation from an oxygenator during CPB may reduce blood loss and need for blood products after cardiac surgery.

## INTRODUCTION

Bleeding occurs in many patients following cardiac surgery under cardiopulmonary bypass (CPB). Massive blood loss and subsequent blood transfusion are associated with high morbidity and mortality.^1–5^ Mounting evidence suggests that the primary cause of blood loss after cardiac surgery is CPB-induced platelet activation.^6–9^ Activated platelets upregulate their surface expression of adhesion molecules such as P-selectin and glycoprotein (GP) IIb/IIIa^6,7,10,11^ and release platelet-specific proteins from alpha granules, such as platelet factor 4, which can induce progressive platelet dysfunction.^7,8^ This dysfunction is observable as weakened platelet aggregation in response to agonists such as collagen^10^ and adenosine diphosphate (ADP).^12^ For example, chest tube drainage after surgery has been associated with impaired collagen-induced platelet aggregation.^13^ Risk of massive blood loss can be reduced by increasing the numbers of collagen-activated functional platelets.^6^

Recent work suggests that volatile anesthetics, especially sevoflurane,^14–17^ can attenuate platelet activation much more than the anesthetics isoflurane and desflurane. Sevoflurane inhalation significantly delayed platelet aggregation in a clinical trial involving 30 patients scheduled to undergo minor elective surgery such as knee arthroscopy or hand surgery.^14^ In an in vitro study involving blood samples from healthy volunteers, low-dose sevoflurane incubation for 1 hour reduced ADP-induced platelet aggregation.^15^ In small samples of healthy subjects^16^ and patients on maintenance anesthesia,^17^ sevoflurane reduced the formation of platelet-leukocyte conjugates. How the anesthetic exerts these effects is unclear. It may do so by inhibiting cyclooxygenase activity and therefore TXA2 formation,^18^ as well as by inhibiting expression of GPIIb/IIIa^16^ and P-selectin^15,17^ on the platelet surface. These studies raise the possibility that sevoflurane inhalation during CPB may reduce the volume of postoperative blood loss and the need for blood products. On the other hand, the effects of sevoflurane on platelets may last up to 24 hours;^16^ since hemostasis requires platelet activation, this prolonged effect may actually raise the risk of postoperative bleeding.^19^

Whether sevoflurane inhalation during cardiac surgery reduces or increases bleeding is unknown. Therefore, we undertook this prospective, randomized, controlled pilot study to examine the effect of sevoflurane inhalation on bleeding.

## METHODS

### Patients

This prospective, randomized, controlled, single blind study was performed in West China Hospital, Sichuan University. The study protocol was approved by the ethics review board of West China Hospital, and all patients gave written informed consent before participation.

Adult patients between 18 and 65 years old who were scheduled to undergo coronary artery bypass grafting and/or valve replacement surgery under CPB at our hospital between November 2014 and May 2015 were eligible for enrollment. Patients were excluded if they had a history of emergency surgery, repeat surgery, abnormal coagulatory function, or hematologic disorders; a platelet count < 50 × 10^9^ or >300 × 10^9^/L, hemoglobin concentration <10 g/dL, renal (serum) creatinine concentration >132.6 mol/L, or history of liver dysfunction with prolonged prothrombin time and/or elevated international normalized ratio. Patients were also excluded if they were already enrolled in other clinical trials.

Patients on aspirin or antiplatelet therapy were enrolled in our trial only if such therapy was halted at least 5 or 7 days, respectively, before surgery. This is in accordance with international guidelines.^20^ Patients exited the study if there was a deviation from the planned surgical procedures, confirmed surgical bleeding or death within 12 hours after surgery.

### Anesthesia and CPB Priming

Anesthesia was performed using procedures that are standard at our hospital.^21^ Briefly, anesthesia was induced using sufentanil supplemented with midazolam and nondepolarizing muscle relaxants, then maintained with propofol and inhaled sevoflurane. After median sternotomy and systemic heparinization (375 U/kg), an aortic cannula and 2 venous cannulas were inserted into the distal ascending aorta and upper and inferior vena cava in order to set up CPB. The bypass equipment included a roller pump, membrane oxygenator (Medtronic, Minneapolis, MN), and tubing system. CPB was primed using 500 mL lactated Ringer solution, 1000 mL succinylated gelatine injection, and 3750 U heparin. Upon CPB completion, residual pump blood was collected into a blood bag containing sodium citrate, neutralized by protamine, and returned to the patient.^21^ All patients received tranexamic acid (2 g) within 30 minutes after CPB. After bleeding stopped, 2 drainage tubes were placed into the pericardium before chest closure in order to drain blood effusion.

### Study Design

Using a random number table, patients were randomized into 2 groups before CPB began. Patients in the control group received intravenous anesthetics throughout the bypass procedure. Patients were continuously infused with propofol (3–6 mg/kg/hour) and Sufentanil (0.5-1 mcg/kg/hour); midazolam a muscle relaxants were given when necessary. Patients in the sevoflurane group received the same intravenous anesthetics, as well as sevoflurane (0.5–1.0 vol%) from the oxygenator. The perfusionist knew the patient allocation, but the anesthesiologist, surgeon, postoperative clinical staff, and data analysts were blinded to group assignment.

Patients in the sevoflurane group received sevoflurane from a vaporizer connected to an air blender, which was connected in turn to an oxygenator. After initiation of CPB, patients received 0.5 vol% sevoflurane by inhalation from the oxygenator. To maintain mean arterial pressure within 20% of its baseline value, sevoflurane dose was adjusted within the range 0.5 to 1 vol% and a vasopressor was administered as necessary.

We estimated the minimal acceptable sample size to be 70 patients in each of the 2 CBP groups in order to have a 75% likelihood of detecting a 100-mL difference in total blood loss between the 2 groups, assuming a standard deviation of 200 mL and a 2-sided alpha level of 5%.

### Outcomes

The primary outcome was blood loss from the chest tube within 24 hours after cardiac surgery. One secondary outcome was the need for transfusion of blood products, defined as packed red blood cells (PRBCs), fresh frozen plasma (FFP), platelets, prothrombin complex, fibrinogen, or cryoprecipitate. Indications for blood product transfusion were the same as those routinely used at our hospital.^21^ Briefly, PRBC was added to the circuit as needed to maintain hemoglobin levels >7 g/dL during CPB, and it was given to patients in the operating room when hemoglobin levels were <8 g/dL and to patients in the intensive care unit (ICU) when hemoglobin levels were <9 g/dL. FFP was given in the operating room at the discretion of the anesthesiologist or surgeon based on bleeding in the surgical field. FFP was given in the ICU whenever mediastinal drainage was >150 mL/hour. Cryoprecipitate (25–30 mL), prothrombin complex and/or fibrinogen were given if mediastinal drainage was >100 mL/hour after activated clotting time had returned to normal. Platelets were given in the operating room at the discretion of the anesthesiologist or surgeon, and/or in the ICU when postoperative platelet count was <50 × 10^9^/L.

Another secondary outcome was the occurrence of adverse events, which might arise from systemic inflammatory response and/or blood product transfusion. Such events included respiratory failure, heart failure, renal failure, nervous system damage, and any cause of death within 30 days after cardiac surgery. Organ dysfunction was defined as described previously.^22^ Briefly, respiratory failure was defined as PaO_2_/FiO_2_ ≤ 100 mm Hg with PEEP ≥ 5 cm H_2_O in the absence of cardiac dysfunction. Renal dysfunction was defined as postoperative serum creatinine level >177 μM and an increase >62 μM from preoperative baseline. Heart failure was defined as congestive heart failure or acute myocardial infarction. Patients experiencing failure of >2 organs were classified as having multiple organ dysfunction.

### Statistical Analysis

Data were analyzed using SPSS 13.0 (IBM, Chicago, IL). Normally distributed continuous data were expressed as mean ± standard deviation and were compared between groups using one-way analysis of variance. Non-normally distributed continuous data were expressed as the median (range), and inter-group differences were assessed for significance using the Kruskal–Wallis test. Categorical data were expressed as a percentage, and intergroup differences were assessed using the Chi-squared or Fisher exact tests, with continuity correction when appropriate. Pearson correlation analysis was used to assess a possible association between platelet count and blood loss after cardiac surgery. Univariate and multivariate logistic regression were performed to identify possible associations between sevoflurane and each predefined outcome. In these analyses, odds ratios were used to estimate the predicted change in risk for a unit increasing in the predictor variable. Regression was performed with and without adjustment for age, body weight, New York Heart Association classification, hypertension, diabetes, platelet count, antiplatelet drugs, coagulatory function, surgery type, and CPB duration. The threshold of significance was defined as *P* < 0.05.

## RESULTS

**FIGURE 1:**
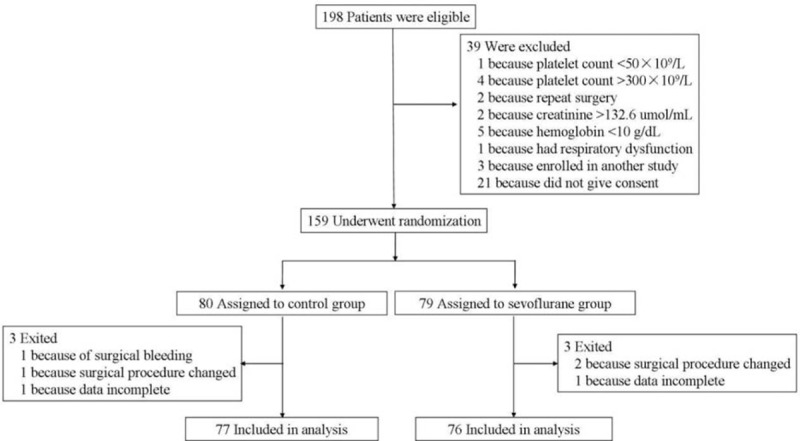
Patient selection, randomization, and analysis.

Of 198 eligible patients, 39 were excluded and 6 exited the study, leaving 153 in the final analysis (Figure 1). The 2 groups were similar in terms of duration of surgery and CPB, but the sevoflurane group showed marginally higher platelet count and plasma fibrinogen concentration (Table 1).

**TABLE 1:**
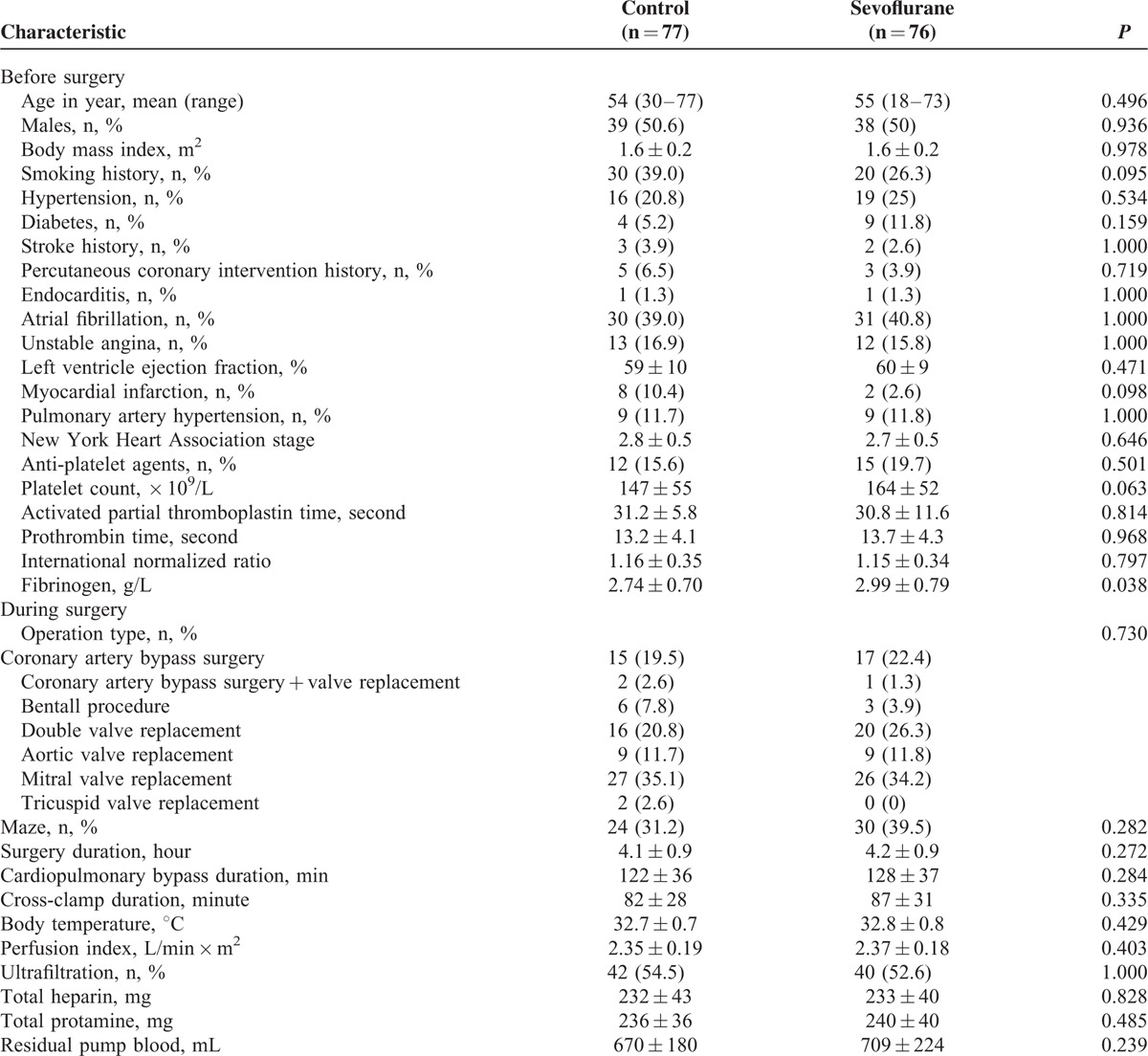
Characteristics of Cardiac Surgery Patients Who Received Inhaled Sevoflurane or not During Cardiopulmonary Bypass

### Sevoflurane Inhalation During CPB Reduced Blood Loss After Surgery

**FIGURE 2:**
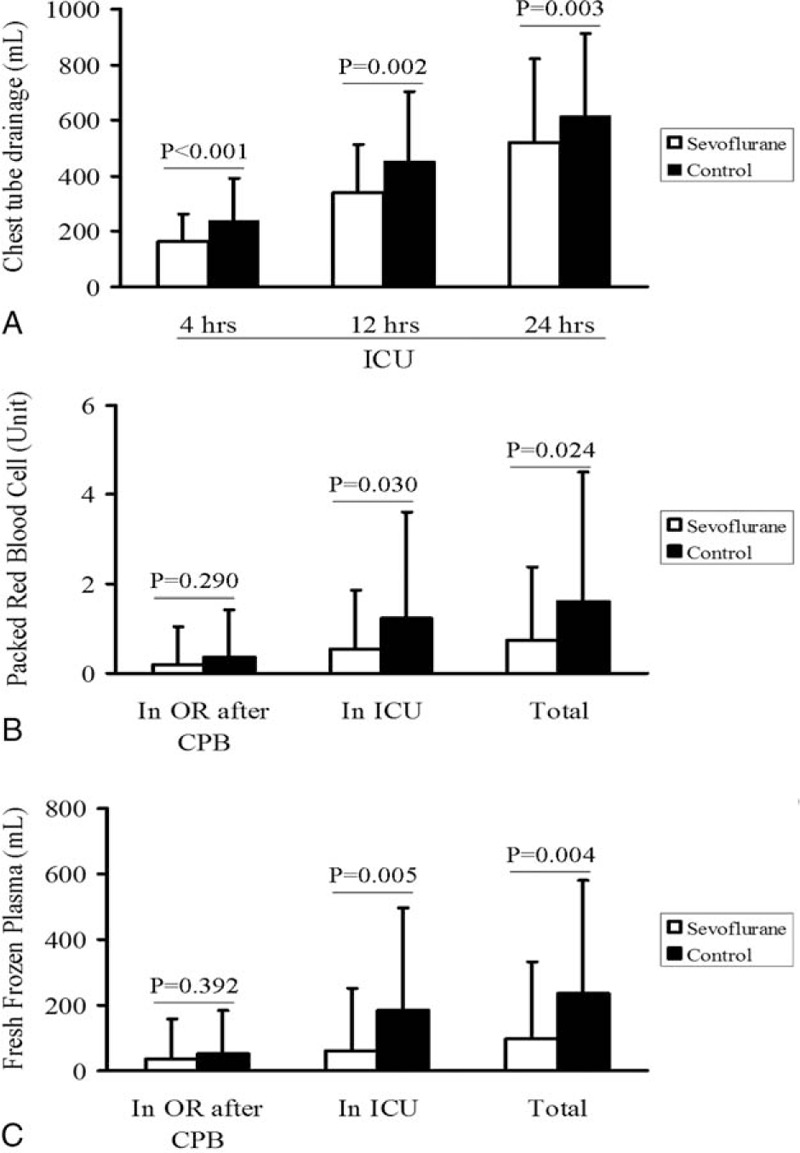
Postoperative outcomes in patients who received inhaled sevoflurane or not during cardiopulmonary bypass. (A) Chest tube drainage, (B) packed red blood cell transfusion, and (C) fresh frozen plasma transfusion. “In OR after CPB” refers to the interval between when patients were weaned off CPB and when they arrived in the intensive ICU. CPB = cardiopulmonary bypass, ICU = intensive care unit, OR = operation room.

Chest tube drainage across all patients was 556 ± 196 mL within 24 hours after surgery, and it was significantly lower in the sevoflurane group after 4, 12, and 24 hours in the ICU (Figure 2A). Drainage volume was 48% lower in the sevoflurane group (78 mL) at 4 hours, and 33% lower (113 mL) at 12 hours. We defined drainage >700 mL per 24 hours as massive blood loss, based on the fact that the first-quartile drainage volume was 380 mL and the third-quartile volume was 695 mL. The proportion of patients experiencing massive blood loss was smaller in the sevoflurane group than in the control group (Table 2). Since this result may be influenced by intergroup differences in several factors that can increase postoperative bleeding, we estimated risk of massive blood loss after adjusting for body weight, platelet count, antiplatelet therapy, coagulatory function, type of surgery, and CPB duration. This analysis indicated that sevoflurane significantly reduced risk of massive blood loss (Table 3).

**TABLE 2:**
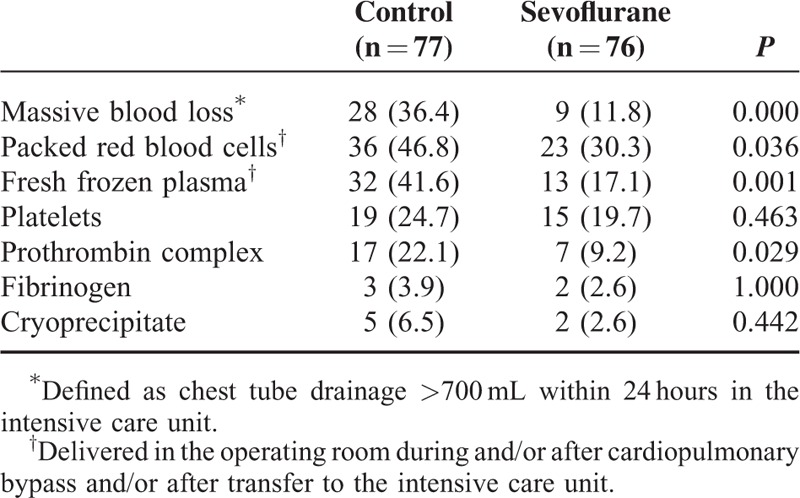
Rates of Massive Blood Loss and Blood Product Use After Cardiac Surgery in Patients Who Received Inhaled Sevoflurane or Not During Cardiopulmonary Bypass (n, %)

**TABLE 3:**
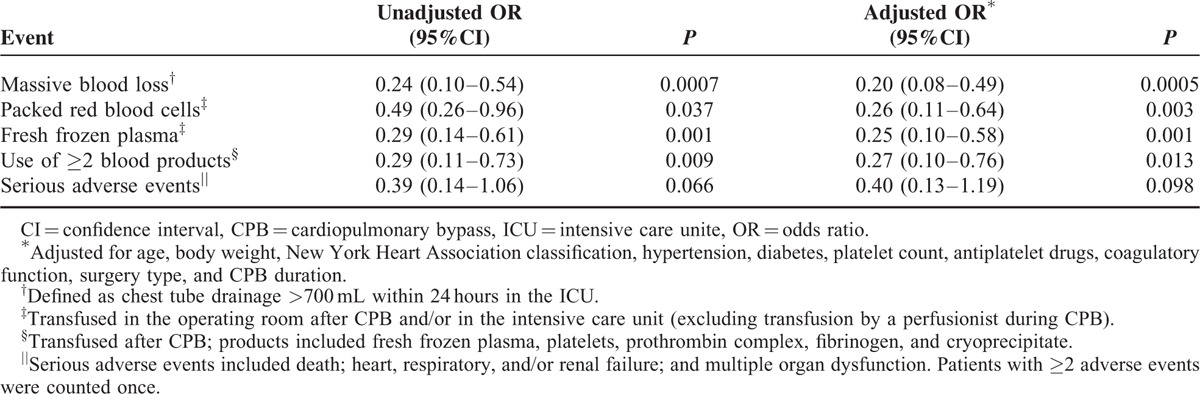
Risk of Massive Bleeding and Need for Blood Products After CPB

### Sevoflurane Decreased Use of Blood Products After Surgery

**FIGURE 3:**
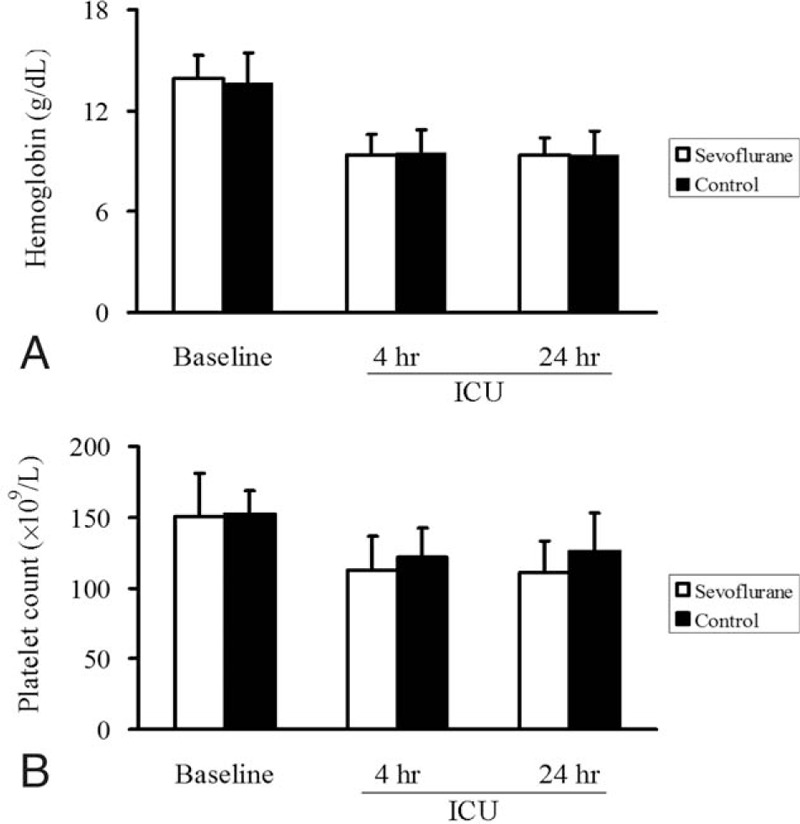
Changes in hemoglobin levels (A) and platelet counts (B) between baseline and after cardiac surgery in patients who received inhaled sevoflurane or not during cardiopulmonary bypass. ICU = intensive care unite.

The 2 CPB groups showed similar hemoglobin levels during surgery (Figure 3A). Across all 153 patients, 37 (24.1%) received PRBC transfusions, while 45 (29.4%) received FFP. Approximately 1-quarter of PRBC transfusions (59 units, 25%) and a handful of FFP transfusions (800 mL, 3%) were given by a perfusionist during CPB. The remaining 3-quarters of PRBC transfusions and 97% of FFP transfusions were administered after CPB; in fact, 57% of all PRBC and 71% of all FFP transfusions were administered in the ICU.

During CPB, the rate and volume of PRBC transfusions were similar between the control group (11 of 77 patients, 0.42 ± 1.06 units) and sevoflurane group (12 of 76 patients, 0.36 ± 0.86 units; *P* = 0.702). One patient in each group received an FFP transfusion during CPB (*P* = 0.533).

The rates of PRBC and FFP transfusions in the operating room after CPB were marginally lower in the sevoflurane group than in the control group, and the corresponding rates in the ICU were significantly lower in the sevoflurane group (Figure 2B and C). As a result, a smaller proportion of sevoflurane patients received PRBC and/or FFP transfusions after CPB (Table 3).

All transfusions of platelets, prothrombin complex, fibrinogen, and cryoprecipitate were administered after CPB. Rates of these perfusions were similar between the 2 groups, except for the rate of prothrombin complex transfusion, which was lower in the sevoflurane group (Table 2). Similarly, the total amount of prothrombin complex transfused was smaller in the sevoflurane group (39 ± 126 vs 94 ± 200 units, *P* = 0.049).

**FIGURE 4:**
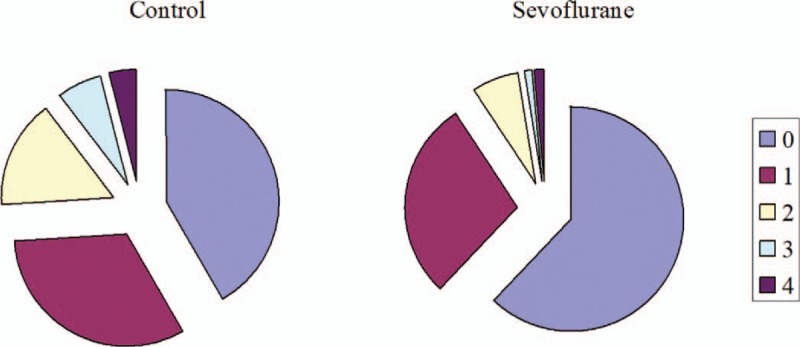
Proportions of patients receiving transfusions of 0 to 4 blood products during and after cardiac surgery. Patients had been randomized to receive inhaled sevoflurane or not during cardiopulmonary bypass. Blood products included fresh frozen plasma, platelets, prothrombin complex, fibrinogen, and cryoprecipitate.

Patients who experienced massive blood loss after CPB represented 24% of our patient population but accounted for a much higher proportions of transfusions: PRBC, 51%; FFP, 64%; platelets, 40%; prothrombin complex, 63%; fibrinogen, 53%; and cryoprecipitate, 91%. Since the control group contained a higher proportion of patients with massive blood loss, we examined whether the control group required a larger amount of blood products (Figure 4). Indeed, the sevoflurane group was at significantly lower risk of needing complex blood products for hemostasis (unadjusted odds ratio [OR] 0.40, 95% confidence interval [CI] 0.21–0.74, *P* = 0.004; adjusted OR 0.34, 95%CI 0.17–0.68, *P* = 0.002). Similarly, the sevoflurane group was at significantly lower risk of requiring ≥2 types of blood products (Table 3).

To analyze the effects of sevoflurane on hemostasis, we determined platelet counts before surgery and at 4 and 24 hours after surgery. Platelet counts were similar between the 2 groups at all-time points (Figure 3B).

### Sevoflurane Did Not Reduce Postoperative Adverse Events

Although sevoflurane was associated with a significantly shorter ICU stay (Table 4), it did not affect duration of intubation or hospitalization. Similarly, it did not significantly affect the rate of serious or other adverse events within 30 days after cardiac surgery (Table 4). Risk of serious adverse events within 30 days was similar between the 2 groups (Table 3).

**TABLE 4:**
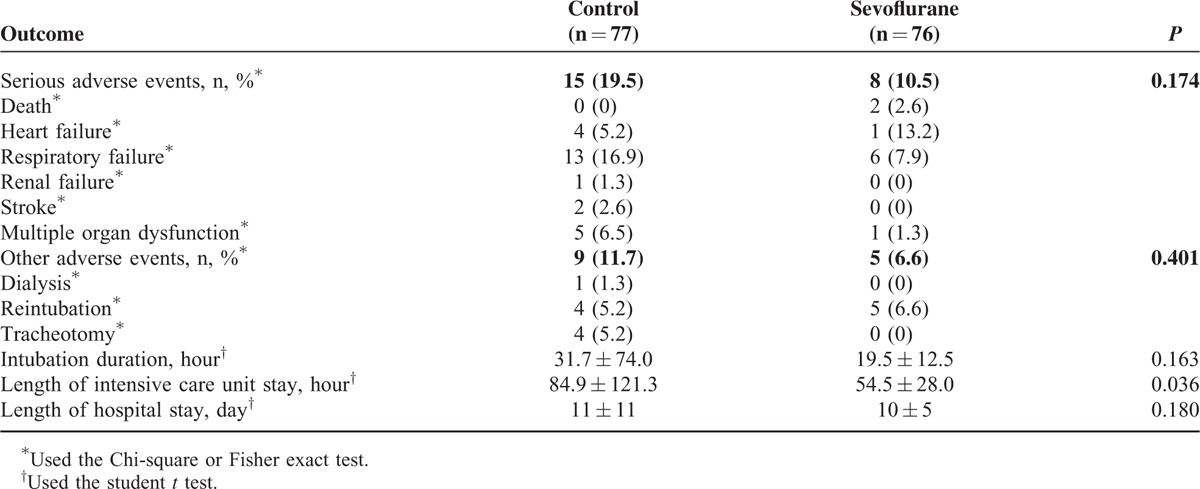
Outcomes at 30 Days After Cardiac Surgery in Patients Who Received Inhaled Sevoflurane or not During Cardiopulmonary Bypass

## DISCUSSION

Previous studies suggest that 15% to 20% of cardiac patients consume 80% of all blood products used during operations,^20^ and in our cohort, the quartile of cardiac patients who experienced massive blood loss accounted for more than half of blood product use. This highlights the need to identify simple, effective strategies to reduce blood loss during cardiac surgery. We provide evidence in this pilot study that inhalation of 0.5 to 1 vol% sevoflurane from an oxygenator during CPB reduces postoperative blood loss, especially massive blood loss, as well as the need for blood products.

Volatile anesthesia is a double-edged sword, primarily because of dose-dependent effects.^23^ Although sevoflurane concentrations of 0.5 to 1 vol% exert strong antiaggregatory effects,^18^ concentrations of 1.71 to 3.42 vol% may increase P-selectin expression on the surface of platelets, inducing their binding to leukocytes.^24^ High concentrations of sevoflurane also affect hemodynamics; indeed, our pilot study with 121 patients not involved in the present work showed that inhalation of 1 to 1.5 vol% sevoflurane from an oxygenator increased the requirement for vasopressors (to >5 mg of metaraminol per case) in order to maintain mean arterial pressure above 50 mm Hg. This may reflect the combination of sevoflurane with intravenous anesthesia during CPB. In contrast, a low concentration of 0.5 to 1 vol% in our pilot study significantly reduced the requirement for metaraminol during CPB (to <3 mg per case). Therefore, we used low sevoflurane concentrations of 0.5 to 1 vol% in the present work.

In our study, blood loss within 24 hours after cardiac surgery was as high as 550 mL, yet chest tube drainage was significantly lower in the sevoflurane group. This contrasts with previous studies of noncardiac surgery suggesting that sevoflurane increases risk of postoperative bleeding.^19,25^ This discrepancy may reflect the different types of surgery involved, as well as the small samples in those previous studies. The proportion of patients needing PRBC transfusion in our sevoflurane group was as low as 30.3%, suggesting that low-dose sevoflurane reduces the requirements for PRBC transfusion to a similar extent as aprotinin and tranexamic acid.^26,27^ In addition, we found that low-dose sevoflurane did not increase the rate of adverse events after surgery, in contrast to aprotinin and tranexamic acid.^26,27^

The main reason for bleeding after cardiac surgery is CPB-induced clotting defects involving decreased coagulation, fibrinolysis, and thrombocytopenia. Correcting these defects often necessitates transfusion of FFP or other blood products. Among our entire cohort, 29% received FFP, 22% received platelets, and 17.6% of patients received >2 types of blood products when bleeding in the surgical field did not improve in the operating room, or when blood loss remained >150 mL/hour in the ICU after FFP transfusion. Nevertheless, the sevoflurane group received less FFP and prothrombin complex than the control group, and the proportion of patients receiving ≥2 types of blood products was lower in the sevoflurane group. These results suggest that low-dose sevoflurane inhalation during CPB can improve hemostasis.

The ability of sevoflurane to reduce blood loss may reflect an influence on platelet counts and/or platelet function. The 2 groups in our study showed similar platelet counts, leading us to suggest that sevoflurane may have influenced the proportion of active platelets. This possibility is consistent with increasing evidence that platelet function, rather than platelet count, plays a more important role in coagulation. Platelet count cannot be used to evaluate platelet-involved clotting,^28^ and some investigators recommend platelet transfusion for patients with clotting time >40 seconds even when platelet counts are as high as 100 × 10^9^/L.^24^ One study reported that total platelet counts in cardiac surgery patients were similar between those experiencing small or large blood loss, but that those experiencing small blood loss showed significantly higher functional platelet counts.^6^ Patients with impaired platelet function during CPB are more likely to require intraoperative transfusions than patients with better platelet function,^8,9^ and platelet dysfunction appears to predict high blood loss after operation.^6,29^

In this way, the results of our preliminary study lead us to propose that sevoflurane inhalation preserves the function of platelets by inhibiting their CPB-induced activation. Although this hypothesis would explain our results and those of a previous study,^30^ it begs the question: why does not sevoflurane increase blood loss? The answer may relate to the role of platelets in coagulation. Intraoperative transfusion and 24-hour chest tube drainage are associated with platelet aggregation induced by collagen and thrombin receptor-activating peptide, but not with aggregation induced by ADP.^6,13^ Studies in vitro suggest that sevoflurane inhibits ADP-induced platelet aggregation, but not thrombin- or collagen-induced aggregation.^31^ This may also help explain why blood loss after endoscopic sinus surgery was similar for patients anesthetized with inhaled sevoflurane or with intravenous propofol.^30^

Our pilot study demonstrates the potential of low-dose sevoflurane to reduce blood loss following cardiac surgery, which should be verified and extended in larger studies. These future studies should avoid the limitations of the present work by including patients from multiple institutions. In addition, future work may not wish to follow our anesthesia protocol, which calls for low-dose sevoflurane inhalation before and after CPB. Future studies may also wish to impose clear, predetermined thresholds for platelet transfusion, since lack of data on platelet counts and function in the operating room can make the decision for platelet transfusion arbitrary. Future work may also wish to record blood loss after weaning from CPB to gain a more complete picture of the effects of low-dose sevoflurane on postoperative blood loss.

Future work should examine directly the hypothesis that low-dose sevoflurane reduces blood loss by inhibiting platelet activation and preserving platelet function after CPB. This can be queried by examining agonist-induced platelet aggregation and measuring levels of coagulation factors. Regardless of the mechanism, if inhalation of 0.5 to 1.0 vol% sevoflurane from an oxygenator during CPB can be confirmed to reduce blood loss, it may have significant clinical impact.
